# Association Between Serum Follicle‐Stimulating Hormone Levels and Risk of Elevated Blood Pressure and Hypertension Among Postmenopausal Women: A Longitudinal Population‐Based Study

**DOI:** 10.1002/hsr2.71325

**Published:** 2025-10-15

**Authors:** Fahimeh Ramezani Tehrani, Faegheh Firouzi, Ramin Farrokhi, Marzieh Saei Ghare Naz, Maryam Mousavi, Fereidoun Azizi, Samira Behboudi‐Gandevani

**Affiliations:** ^1^ Reproductive Endocrinology Research Center, Research Institute for Endocrine Molecular Biology, Research Institute for Endocrine Sciences Shahid Beheshti University of Medical Sciences Tehran Iran; ^2^ Foundation for research & Education Excellence Vestavia Hills Alabama USA; ^3^ Endocrine Research Center, Research Institute for Endocrine Disorders, Research Institute for Endocrine Sciences Shahid Beheshti University of Medical Sciences Tehran Iran; ^4^ Faculty of Nursing and Health Sciences Nord University Bodø Norway

**Keywords:** elevated blood pressure, follicle stimulating hormone, hypertension, post‐menopausal women

## Abstract

**Background and Aims:**

Postmenopausal‐women experience a rise in follicle‐stimulating hormone (FSH) levels, yet its association with the risk of elevated blood pressure (BP) and hypertension (HTN) remains unclear despite the increased cardiovascular risk in this population. This study first investigated the association between serum FSH levels and both the prevalence and risk of elevated BP and HTN in a long‐term, population‐based cohort of menopausal‐women. Second, we assessed whether FSH levels were associated with longitudinal trajectories of systolic and diastolic blood pressure (SBP and DBP) over time.

**Methods:**

Data from 934 post‐menopausal‐women (aged ≥ 40) in the population‐based Tehran Lipid and Glucose were analysed over six phases between 2002 and 2021. Logistic regression assessed the association baseline FSH and prevalent HTN7elevated BP. Cox proportional‐hazards models estimated incident HTN7elevated BP risk. Linear mixed models evaluated SBP and DBP trajectories by FSH level, adjusting for demographic, metabolic, and lifestyle factors.

**Results:**

Among 934 postmenopausal women, mean (SD) age: 58.64 (6.72) years, 43.3% had HTN and 32.8% had elevated BP at baseline. Over 4868 person‐years of follow‐up, 295 women (60.6 per 1000 person‐years) developed HTN, and over 2034 person‐years, 134 (65.9 per 1000 person‐years) developed elevated BP. Baseline FSH levels, whether analyzed categorically or continuously, showed no significant association with the prevalence of HTN (fully‐adjusted OR: 0.87, 95% CI: 0.60–1.27) or elevated BP (fully‐adjusted OR: 0.85, 95% CI: 0.55–1.30), nor with the incidence of HTN (fully‐adjusted HR: 0.975, 95% CI: 0.737–1.288) or elevated BP (fully‐adjusted HR: 1.11, 95% CI: 0.74–1.68). Longitudinal analyses revealed significant changes in SBP and DBP over time, but these trajectories were not influenced by baseline FSH levels.

**Conclusion:**

We found no significant association between baseline serum FSH levels and the prevalence or incidence of elevated BP and HTN, nor with longitudinal changes in systolic or diastolic blood pressure, despite significant BP trajectory changes over the follow‐up period.

## 1 Introduction

Hypertension (HTN) remains a leading global health challenge, contributing significantly to cardiovascular morbidity and mortality, particularly among aging populations [1, 2]. It is estimated that the prevalence of HTN among adults aged 30–79 years globally was around 30%–40% [1, 3].

The menopausal transition marks a critical period for cardiovascular health, during which post‐menopausal women experience a more pronounced rise in HTN prevalence compared to age‐matched men [4]. While traditional risk factors such as obesity, physical inactivity, and genetic predisposition contribute to this trend, the hormonal milieu of menopause may play a pivotal role in modulating these risks [5]. The complex interplay of hormonal changes, aging, and metabolic alterations following menopause further exacerbates the risk of HTN in this population (3).

It has been shown that the prevalence of HTN among post‐menopausal women is projected to increase to 40%, a trend attributed not only to aging but also to changes in sex hormones [4, 6, 7]. The transition to menopause is characterized by profound hormonal shifts, including a decline in estrogen levels and a compensatory rise in follicle‐stimulating hormone (FSH). Emerging evidence suggests that these hormonal changes may contribute to the pathophysiology of HTN, yet the precise mechanisms remain poorly understood.

FSH, a gonadotropin hormone secreted by the anterior pituitary, is traditionally recognized for its role in regulating ovarian function and follicular development during the reproductive years [8, 9]. In post‐menopausal women, FSH levels rise significantly due to the loss of negative feedback from ovarian estrogen production [10]. Emerging evidence suggests that elevated FSH levels may have extra‐gonadal effects, influencing metabolic and cardiovascular homeostasis [11, 12]. For instance, FSH has been shown to interact with receptors in non‐ovarian tissues, including vascular endothelial cells and adipocytes, potentially contributing to inflammation, insulin resistance, and dyslipidemia as key risk factors for HTN [13, 14, 15]. Furthermore, some studies have demonstrated that FSH may induce oxidative stress and free radical generation, further implicating its role in blood pressure regulation [12, 16]. As such, it is suggested that FSH may increase renin production involved in the regulation of blood pressure in post‐menopausal women [17]. Despite these findings, the association between serum FSH levels and HTN risk in post‐menopausal women has not been comprehensively explored in large‐scale, population‐based studies.

To address this knowledge gap, we conducted a long‐term, population‐based study to investigate the association between serum FSH levels and both the prevalence and incidence of elevated BP and HTN among postmenopausal women. As a secondary objective, we examined whether baseline FSH levels were associated with longitudinal trajectories of SBP and DBP over time.

## 2 Material and Methods

In this cohort study, subjects were recruited from among participants of the Tehran Lipid and Glucose Study (TLGS), an ongoing population‐based cohort, initiated in 1998 to investigate the prevalence and risk factors of non‐communicable diseases. In summary, in TLGS, a total of 15,005 individuals, aged ≥ 3 years, were followed at intervals of 3 years, to obtain data on demographics, anthropometric, reproductive, hormonal and metabolic characteristics, general physical examinations and laboratory measurements. For the purpose of the present study, we used TLGS data from six follow‐ups (1st: 2002–2005, 2nd: 2005–2008, 3rd: 2008–2011, 4th: 2011–2014, 5th: 2014–2018, and 6th: 2018–2021).

At each visit, subjects were assessed for clinical, and anthropometric, and biochemical parameters by a trained interviewer. Body weight was measured with at least clothed using a digital scale (Seca 707, Seca GmbH) and rounded to the nearest 100 g. Likewise, height was measured without shoes in standing position and normal posture of shoulders with a tape measure. Body mass index (BMI) was calculated using formula [weight in kilograms (kg) divided by height squared (m^2^)]. Waist circumference was measured with an un‐stretched tape measure at the level of the umbilicus, without any pressure to the body surface. Hip circumference was measured at the level of the anterior superior iliac spine without any pressure to the body surface. We also measured SBP and DBP twice on the right arm in a seated position using a standard mercury sphygmomanometer after 15 min of rest; then the mean value of these measurements was recorded.

Serum FSH level was measured at first visit, while all other biochemical measurements were conducted at baseline and each follow‐up visit. All blood samples were drawn between 7:00 and 9:00 am after 12 h of overnight fasting; blood analyses were performed at the TLGS research laboratory on the day of blood collection. All sera were stored at –80°C until the time of testing. Serum concentration of FSH was measured ELISA method; the kit was ELISA, IDEAL Tashkis, Tehran, Iran; intra‐ and inter‐assay CVs were 1.9% and 2.5%, respectively. All FSH measurements were taken simultaneously in the same laboratory.

Plasma glucose was measured using an enzymatic colorimetric method with glucose oxidase. Serum concentrations of triglyceride (TG) were assayed using glycerol phosphate. Total cholesterol (TC) was assayed using the enzymatic colorimetric method with cholesterol esterase and cholesterol oxidase. Levels of high‐density lipoprotein cholesterol (HDL‐C) were measured after precipitation of the apolipoprotein B (apo B)‐containing lipoproteins with phosphotungstic acid. We used a modified Friedewald to calculate LDL‐C. All metabolic analyses were performed using related kits (Pars Azmon Inc., Tehran, Iran) and a Selecta 2 autoanalyzer (Vital Scientific, Spankeren, Netherlands). The intra‐ and inter‐assay coefficients of variations (CVs) were both 2.2% for glucose. For both total and HDL‐Cholesterol, intra‐ and inter‐assay CVs were 0.5% and 2%, respectively. Intra‐ and inter‐assay CVs were 0.6% and 1.6% for TG, respectively.

**Figure 1:**
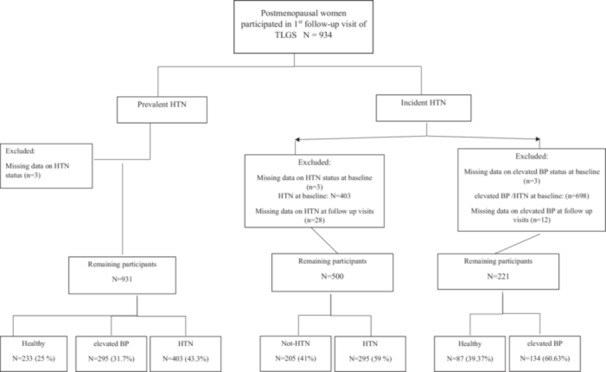
Flow chart of study. HTN, hypertension; TLGS, Tehran Lipid and Glucose Study.

For the present study, we included all postmenopausal women aged ≥ 40 years who met the eligibility criteria and had available FSH measurements at the first follow‐up visit. Two analytical approaches were employed to investigate the association between serum FSH levels and elevated BP and HTN. First, to examine the association between FSH levels and the prevalence of elevated BP and HTN, we included all eligible postmenopausal women, regardless of their hypertensive status at the first visit, excluding only those participants with missing data on elevated BP or HTN (*n* = 3). Second, to assess the association between FSH levels and the incidence of elevated BP and HTN, we excluded participants with missing data on elevated BP (*n* = 15) or HTN (*n* = 31), as well as those who were diagnosed with the respective condition at baseline (elevated BP: *n* = 698; HTN: *n* = 403). A detailed overview of the study population selection process is provided in Figure 1.

### 2.1 Terms Definitions

According to the normal‐based methodology of Altman and Chitty 33 and Royston and Wright 34, age‐specific AMH percentiles were estimated according to the exponential–normal 3‐parameter model. Cut‐off values for categorization of women of each specific age to the age‐specific AMH percentiles have been reported in our previous paper 35. For the purpose of the present study, participants were subclassified to four groups according to quartiles of age‐specific AMH level.

HTN was defined as SBP ≥ 140 mmHg or DBP ≥ 90 mmHg or using anti‐hypertensive medications [18]. Elevated BP was defined as SBP of 120–139 mmHg and/or DBP of 80–89 mmHg without anti‐hypertensive medication [18].

### 2.2 Statistical Analysis

The baseline characteristics of participants according to their FSH values (high vs. low) are presented using the mean and standard deviation or the median and interquartile range (IQR), based on their normal or skewed distribution. The Kolmogorov–Smirnov test is conducted to assess the normality hypothesis. The categorical variables, expressed as percentages. Comparisons between two groups (high and low FSH levels) were assessed with the student's unpaired *t* test and Mann–Whitney test or *χ* ^2^ test, as appropriate. FSH levels were stratified into two subgroups based on a predefined cutoff: high (≥ 40 IU/L) and low (< 40 IU/L).

To evaluate how baseline FSH levels are associated with prevalent HTN at baseline, odds ratios (ORs) and 95% confidence intervals (CIs) are used in logistic regression models. Kaplan–Meier curves were created to illustrate the survival time across FSH categories. Cox proportional‐hazard regression analysis was employed to assess the predictive effect of baseline FSH levels on incident HTN/elevated BP, expressed through hazard ratios (HRs) and 95% CIs. Participants contributed person‐time from study entry until either an HTN/elevated BP diagnosis or their last follow‐up visit (for non‐HTN/elevated BP individuals), whichever occurred first. For the analysis of elevated BP incidence, only participants who were free of both elevated BP and HTN at baseline were included. For the analysis of HTN incidence, only participants without HTN at baseline were considered. Furthermore, subgroup analyses were performed by age, menopause age, BMI, total cholesterol, and triglycerides, and the findings were shown in forest plots.

To investigate the longitudinal relationship between baseline FSH levels and changes in SBP and DBP, linear mixed models with random intercepts are applied to account for repeated measurements within individuals. We evaluated the specified models by adjusting for age and BMI at baseline (Model 2), and by further adjusting for smoking status, total cholesterol, HDL cholesterol, physical activity category, triglycerides, parity, and family history of HTN (Model 3). Scatter plots were also generated to show the relationship between baseline FSH levels and blood pressure values over time.

All analyses are performed in R (version 4.3.2), using packages including dplyr for data processing, survival for Cox regression, table one for summarizing baseline characteristics, lme4 for linear mixed models, and ggplot2 for visualizing blood pressure patterns over time.

## 3 Results

A total of 934 postmenopausal women participated in the study, with a mean age of 58.64 (SD = 6.72) years, mean BMI of 30.04 (SD = 4.53) Kg/m^2^ and a mean age at menopause of 50.50 (SD = 5.45) years. The overall mean baseline serum FSH level was 59.92 (SD = 32.40) mIU/mL. History of smoking and family history of HTN were observed in 5.3%, 22.8%, and 36% of study participants, respectively. 67.5% of participants had adequate to high levels of physical activity. Participants with lower FSH levels demonstrated higher baseline BMI, greater parity, and younger age at menopause compared to those with elevated FSH levels (Table 1). No significant differences were observed for SBP, DBP, lipid Profile, smoking status, physical activity, or family history of HTN across the groups.

**Table 1:** The baseline characteristics of participants, stratified by their baseline follicle‐stimulating hormone values.

Variable	Overall (*n* = 934)	High FSH (*n* = 675)	Low FSH (*n* = 259)	*p* value
Age, years, Mean (SD)	58.64 (6.72)	59.16 (6.22)	57.27 (7.72)	< 0.001
Menopause Age, years, Mean (SD)	50.50 (5.45)	50.96 (4.91)	49.32 (6.53)	< 0.001
BMI, Kg/m^2^ Mean (SD)	30.04 (4.53)	29.66 (4.32)	31.01 (4.92)	< 0.001
Parity Mean (SD)	4.89 (2.04)	5.01 (2.05)	4.60 (1.98)	0.007
SBP, mmHg, Mean (SD)	129.25 (20.90)	128.91 (20.82)	130.14 (21.11)	0.42
DBP, mmHg, Mean (SD)	79.12 (10.70)	78.95 (10.70)	79.58 (10.73)	0.42
HDL Cholesterol, mg/dL, Median (IQR)	41.00 (34, 49)	42 (35, 49)	39 (33, 47)	0.16
Triglycerides, mg/dL, Median (IQR)	170 (122, 232)	167.50 (124, 226)	174 (121, 254.5)	0.27
Total Cholesterol, mg/dL, Mean (SD)	222.79 (43.46)	222.86 (43.12)	222.61 (44.43)	0.94
Family history of HTN, *n* (%)	338 (36.5)	251 (37.5)	87 (33.9)	0.34
Active Physical activity, *N* (%)	619 (67.5)	444 (67.2)	175 (68.4)	0.79
Smoking status, *N* (%)	49 (5.3)	32 (4.8)	17 (6.7)	0.32
Serum FSH, mIU/mL, Mean (SD)	59.92 (32.4)	72.57 (28.88)	26.97 (10.42)	< 0.001

Abbreviations: BMI, body mass index; DBP, diastolic blood pressure; FSH, follicle stimulating hormone; HDL, high‐density lipoprotein; LDL, low‐density lipoprotein; SBP, systolic blood pressure.

### 3.1 Association Between Baseline FSH Levels and Prevalence of HTN and Elevated BP

At baseline, 403 participants (43.3%) were diagnosed with HTN and 305 (32.8%) with elevated BP. Compared with individuals with low FSH levels (< 40 IU/L), those with high FSH levels (≥ 40 IU/L) did not show a significant association with the prevalence of HTN in the unadjusted model (OR: 1.02, 95% CI: 0.76–1.37), nor in the fully adjusted model (OR: 0.87, 95% CI: 0.60–1.27). Similarly, FSH analyzed as a continuous variable was not associated with HTN in any of the models (fully adjusted OR: 0.998, 95% CI: 0.993–1.004; *p* = 0.48). For elevated BP, compared with individuals with low FSH levels (< 40 IU/L), those with high FSH levels (≥ 40 IU/L) did not show a significant association with the prevalence of elevated BP in the unadjusted model (OR: 1.33, 95% CI: 0.96–1.85), or fully adjusted model (OR: 0.85, 95% CI: 0.55–1.30). When FSH was treated as a continuous variable, a marginally significant inverse association was observed in the unadjusted model (OR: 0.994, 95% CI: 0.989–0.999; *p* = 0.048), though this association was not maintained after full adjustment (fully adjusted OR: 0.995, 95% CI: 0.989–1.001; *p* = 0.46) (Table 2).

**Table 2:** Association of follicle‐stimulating hormone (FSH) Levels with prevalence of hypertension and pre‐hypertension diagnosed at baseline.

FSH level	No. of cases	No. of participants	Model 1 OR (95% CI)	Model 2 OR (95% CI)	Model 3 OR (95% CI)
Hypertension					
Low FSH (Ref)*	110	173	Reference	Reference	Reference
High FSH**	293	463	1.02 (0.76–1.37)	0.94 (0.69–1.28)	0.87 (0.60–1.27)
Continuous FSH (IU/L)	403	636	1.001 (0.997–1.005)	1.002 (0.997–1.006)	0.998 (0.993–1.004)
*p*‐value			0.85	0.71	0.48
Pre‐Hypertension					
Low FSH (Ref)^a^	84	147	Reference	Reference	Reference
High FSH^b^	221	381	1.33 (0.96–1.85)	0.857 (0.57–1.27)	0.85 (0.55–1.30)
Continuous FSH (IU/L)	305	528	0.994 (0.989–0.999)	0.994 (0.989–1.0001)	0.9953 (0.989–1.001)
*p*‐value			0.048	0.451	0.458

^a^ < 40 IU/L.

^b^ ≥ 40 IU/L Model 1 Crude model; Model 2 adjusted age and body mass index (BMI); and Model 3 further adjusted for smoking status, total cholesterol, high‐density lipoprotein (HDL) cholesterol, physical activity category, triglycerides, parity, and family history of hypertension.

### 3.2 Association Between Baseline FSH Levels and Incidence of HTN and Elevated BP

For elevated BP, a total of 134 elevated BP cases, who were free of outcome at baseline, were documented over 2034 person‐years of follow‐up. The incidence rate was 65.9 per 1000 person‐years. Supporting Information S1: Table 1 presents the baseline characteristics of women participants, stratified by their elevated BP status at follow‐up. Participants who developed elevated BP exhibited significantly elevated baseline SBP compared to those who remained normotensive (*p* = 0.005). No significant differences were observed between groups for other variables, including age, BMI, cholesterol levels, or lifestyle factors (*p* > 0.05).

**Figure 2:**
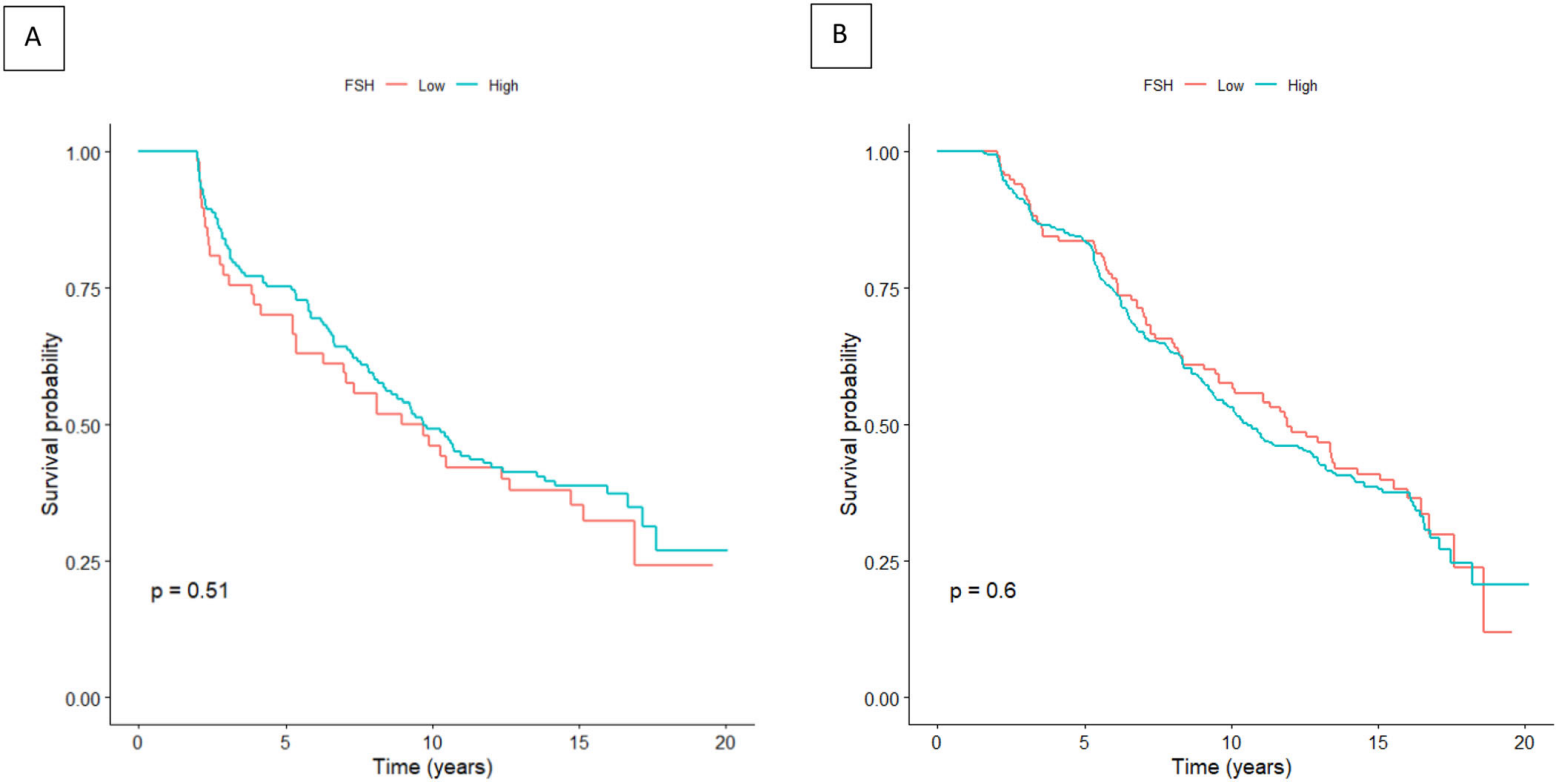
Kaplan–Meier curves depicting (A) elevated BP and (B) HTN stratified by baseline FSH levels (< 40 vs. ≥ 40 IU/L).

The results of Cox proportional‐hazard regression models to evaluate the association between baseline FSH levels and the incidence of elevated BP over the follow‐up period are presented in Table 3. Compared with individuals with low FSH levels (< 40 IU/L), those with high FSH levels (≥ 40 IU/L) showed no significant association with the risk of elevated BP in either the unadjusted (HR: 1.17, 95% CI: 0.79–1.73) or the fully adjusted model (HR: 1.11, 95% CI: 0.74–1.68). The Kaplan–Meier survival analysis curves depicting the incidence of elevated blood pressure demonstrated no statistically significant differences between those with low FSH levels and those with high FSH levels, as evidenced by a log‐rank test yielding a *p*‐value of 0.51 (Figure 2, Panel A). When FSH was analyzed as a continuous variable, no significant association with incident elevated BP was observed in the unadjusted (HR: 1.00, 95% CI: 0.995–1.004) or fully adjusted model (HR: 1.001, 95% CI: 0.996–1.006).

**Table 3:** Association of follicle‐stimulating hormone (FSH) Levels with incidence of pre‐hypertension and hypertension diagnosed during study.

	No. of cases	Person‐year	Incidence rate per 1000 person‐year	Model 1 HR (95% CI)	Model 2 HR (95% CI)	Model 3 HR (95% CI)
Pre‐Hypertension						
Low FSH (< 40 IU/L)	37	516	71.7	Ref	Ref	Ref
High FSH (≥ 40 IU/L)	97	1518	63.9	0.88 (0.60–1.28)	0.87 (0.589–1.30)	0.89 (0.59–1.35)
FSH (IU/L)	134	2034	65.9	1.000 (0.995–1.004)	1.000 (0.995–1.005)	1.001 (0.996–1.006)
*p*‐Value	—	—	—	0.50	0.51	0.60
Hypertension						
Low FSH (< 40 IU/L)	80	1369	58.4	Ref	Ref	Ref
High FSH (≥ 40 IU/L)	215	3499	61.4	1.071 (0.829–1.385)	1.000 (0.997–1.004)	0.975 (0.737–1.288)
FSH (IU/L)	295	4868	60.6	1.000 (0.997–1.003)	1.000 (0.997–1.003)	1.000 (0.996–1.004)
*p*‐Value	—	—	—	0.59	0.87	0.85

*Note:* Mode l: Crude model; Model 2 adjusted for age and body mass index (BMI); and Model 3 further adjusted for smoking status, total cholesterol, high‐density lipoprotein (HDL) cholesterol, physical activity category, triglycerides, parity, and family history of hypertension.

For HTN, a total of 295 cases occurred over 4868 person‐years of follow‐up. The incidence rate was 60.6 per 1000 person‐years. Supporting Information S1: Table 2 presents the baseline characteristics of participants, stratified by their HTN status at follow‐up. Women who developed HTN demonstrated significantly higher mean values for age, BMI, SBP and DBP, parity, and age at menopause compared to their normotensive counterparts (*p* < 0.05). Additionally, a significantly greater proportion of hypertensive women reported a family history of HTN (18.2% vs. 9.8%; *p* = 0.01).

The results of Cox proportional‐hazard regression models to evaluate the association between baseline FSH levels and the incidence of HTN over the follow‐up period are presented in Table 3. High FSH levels (≥ 40 IU/L) were not significantly associated with the risk of developing HTN compared to the low FSH group in either the unadjusted (HR: 1.071, 95% CI: 0.829–1.385) or the fully adjusted model (HR: 0.975, 95% CI: 0.737–1.288). and Kaplan–Meier curves for HTN (Figure 2B) similarly showed no significant difference between FSH groups (log‐rank *p* = 0.60). FSH as a continuous variable also was not significantly associated with HTN in the unadjusted (HR: 1.00, 95% CI: 0.997–1.003) or fully adjusted model (HR: 1.00, 95% CI: 0.996–1.004).

**Figure 3:**
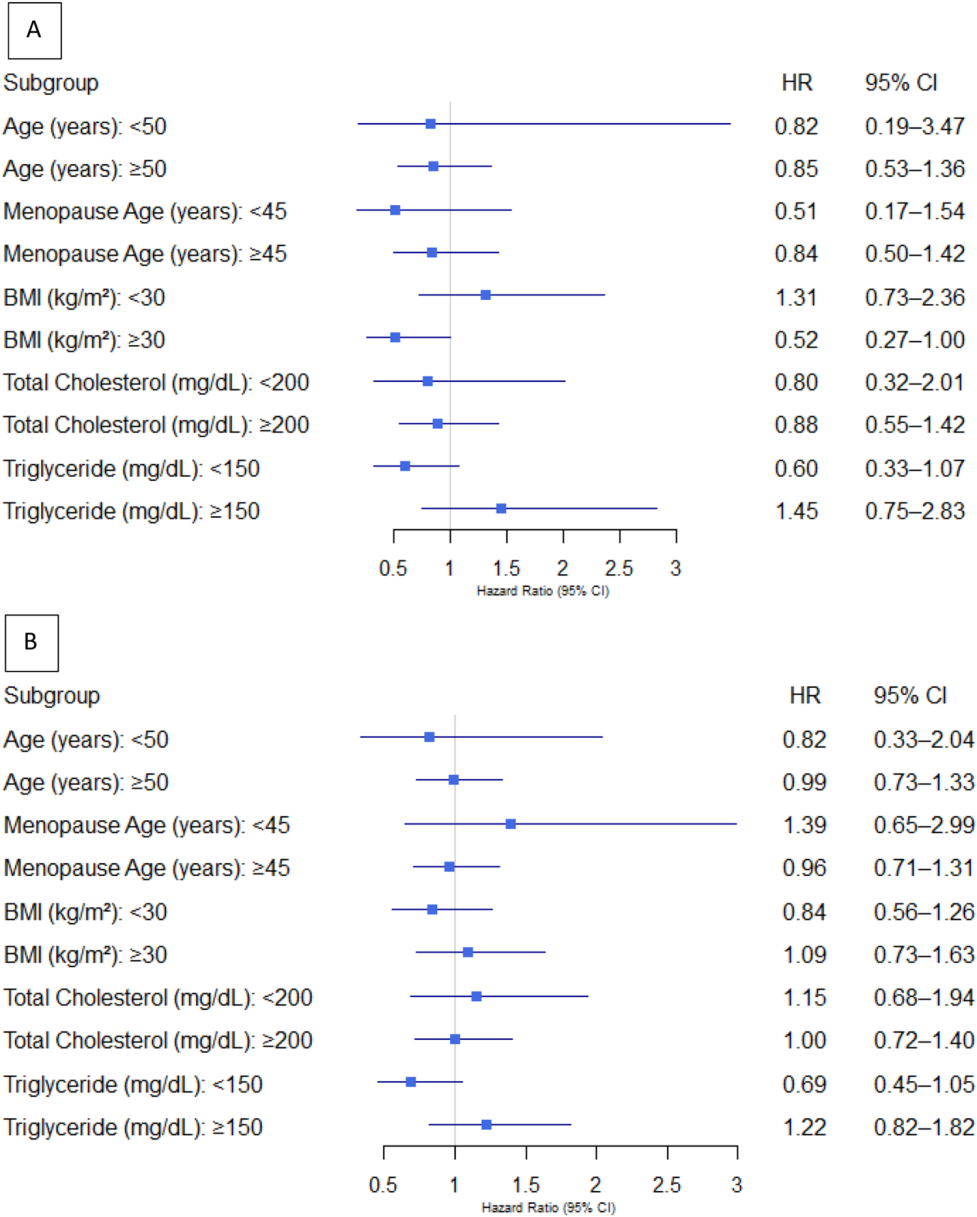
Forest plots depicting subgroup analyses of baseline FSH and incident of (A) elevated BP and (B) HTN.

The findings from the subgroup analyses assessing the relationship between baseline FSH levels and the incidence of HTN and elevated BP are presented in Figure 3. FSH levels were not significantly associated with the risk of developing HTN or elevated BP across subgroups stratified by age, age at menopause, BMI, total cholesterol, and triglyceride levels. These results were consistent with the primary analysis, with the exception of a borderline association observed between elevated BP risk and FSH levels in participants with a BMI of ≥ 30 kg/m^2^.

### 3.3 Longitudinal Association Between Baseline FSH Levels and Trajectories of SBP and DBP Over Time

**Figure 4:**
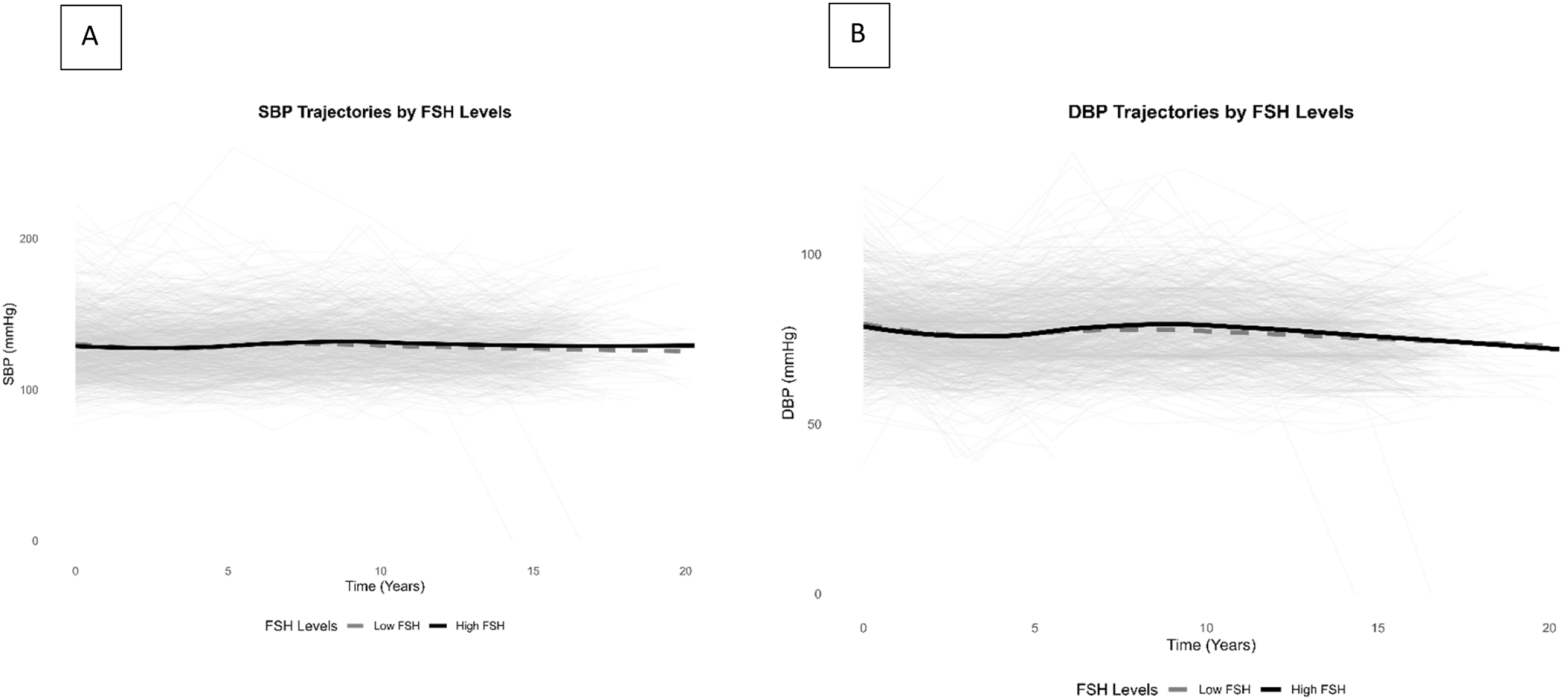
Longitudinal trajectories of systolic blood pressure (A) and diastolic blood pressure (DBP) (B) stratified by dichotomized follicle‐stimulating hormone (FSH) Levels.

Further, we used linear mixed models to assess the longitudinal relationship between baseline FSH levels and trajectories of SBP and DBP over time (Table 4, Figure 4A,B).

**Table 4:** Longitudinal effects of follicle‐stimulating hormone (FSH) on systolic and diastolic blood pressure.

Outcome	Variable	Model 1 estimate (95% CI)	Model 2 estimate (95% CI)	Model 3 estimate (95% CI)
SBP	Intercept	83.55 (74.57–92.53)***	58.56 (47.05–70.07)***	71.80 (58.93–84.67)***
	High FSH (≥ 40 IU/L)	2.10 (−0.47–4.67)	1.26 (−1.32–3.84)	1.53 (−0.95–4.01)
	Time (years)	0.16 (0.06–0.26)**	0.16 (0.06–0.26)**	0.17 (0.07–0.27)**
	High FSH × Time Interaction	−0.09 (−0.29–0.11)	−0.11 (−0.30–0.08)	−0.13 (−0.33–0.07)
DBP	Intercept	79.48 (74.53–84.43)***	63.45 (57.19–69.71)***	67.97 (60.61–75.33)***
	High FSH (≥ 40 IU/L)	0.27 (−1.15–1.69)	−0.24 (−1.64–1.16)	−0.10 (−1.43–1.23)
	Time (years)	−0.12 (−0.18–−0.06)***	−0.12 (−0.18–−0.06)***	−0.12 (−0.18–−0.06)***
	High FSH × Time Interaction	−0.05 (−0.16–0.06)	−0.05 (−0.16–0.06)	−0.08 (−0.19–0.03)

*Note:* SBP: Systolic blood pressure, DBP: Diastolic blood pressure, Model 1: Crude model; Model 2: included age and body mass index (BMI); and Model 3: further adjusted for smoking status, total cholesterol, high‐density lipoprotein (HDL) cholesterol, physical activity category, triglycerides, parity, and family history of hypertension.

*** *p* value = 0.001

** *p* value = 0.001

* *p* value < 0.05.

For SBP, the time effect showed a significant increase of SBP over time in unadjusted (0.16 mmHg/year, 95% CI: 0.06–0.26), and fully adjusted model (0.17 mmHg/year, 95% CI: 0.07–0.27). The interaction term between high FSH (≥ 40 IU/L) and time was not significant, indicating that women with high FSH levels did not experience a significantly different SBP trajectory compared to those with low FSH levels (unadjusted model: −0.09 mmHg/year, 95% CI: −0.29–0.11, and fully adjusted model: −0.13 mmHg/year, 95% CI: −0.33–0.07).

**Figure 5:**
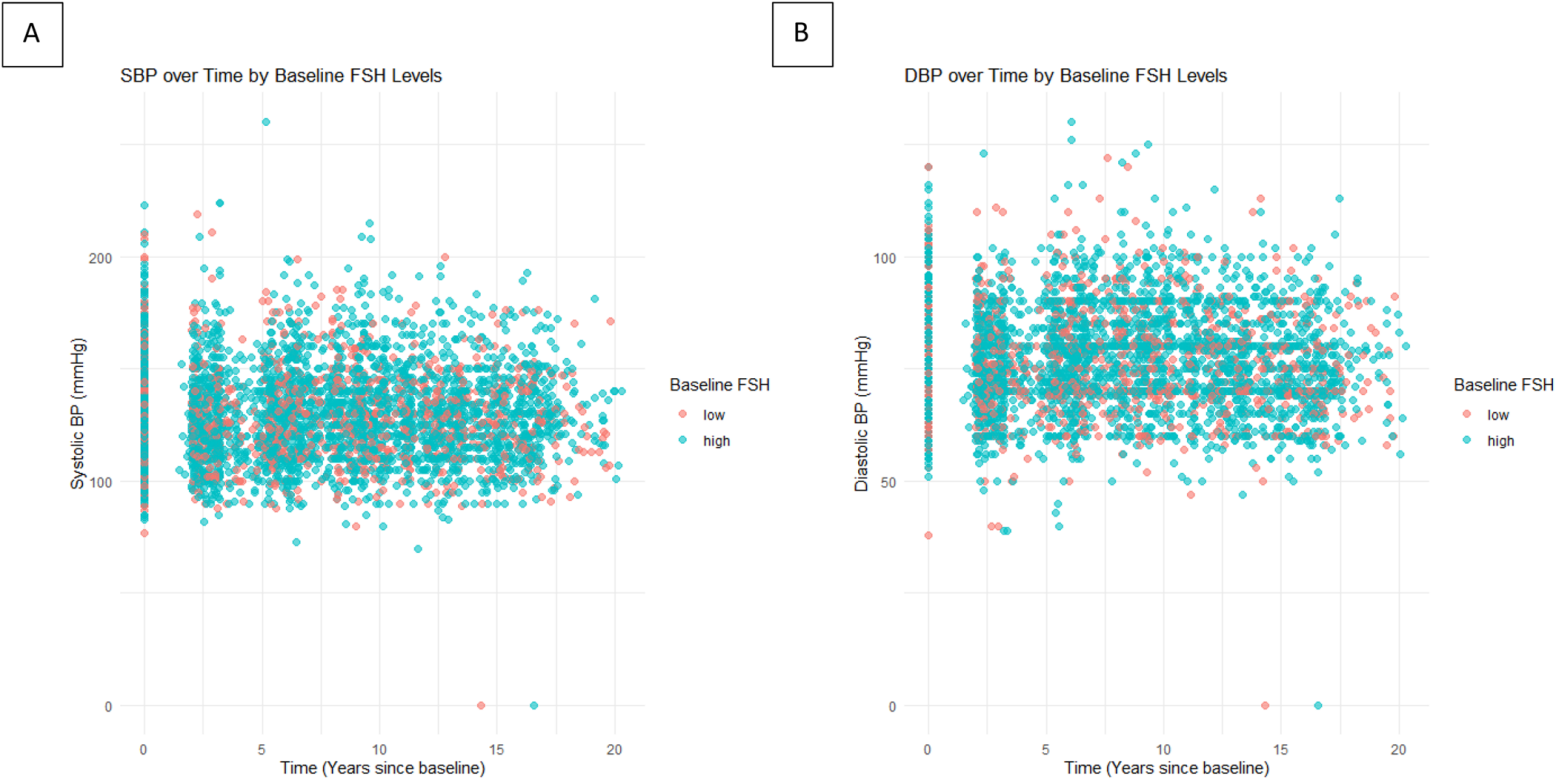
Scatter plot depicting the relationship between baseline FSH levels and (A) SBP or (B) DBP measurements over time.

For DBP, the time effect showed a significant decrease over time in unadjusted (−0.12 mmHg/year, 95% CI: −0.18–−0.06), and fully adjusted model (−0.12 mmHg/year, 95% CI: −0.18 to −0.06), but the interaction term indicating that women with high FSH levels did not experience a significantly different DBP trajectory compared to those with low FSH levels (unadjusted model: −0.05 mmHg/year, 95% CI: −0.16–0.06, and fully adjusted model: −0.08 mmHg/year, 95% CI: −0.19–0.03). Additionally, Figure 5 presents a scatter plot depicting the relationship between baseline FSH levels with SBP and DBP measurements over time.

## 4 Discussion

In this large, population‐based cohort of postmenopausal women, we found (i) no significant association between baseline serum FSH levels and prevalence of elevated BP and HTN; (ii) no significant association between serum FSH levels and the risk of developing elevated BP or HTN over time; and (iii) no significant association between serum FSH levels and longitudinal changes in SBP and DBP; although both SBP and DBP trajectories exhibited significant variation over the follow‐up period, baseline FSH levels did not appear to influence these trends. These findings remained consistent irrespective of whether FSH was analyzed as a categorical or continuous variable and persisted following adjustment for multiple potential confounding factors.

The menopausal transition is characterized by diminished ovarian function and a decline in estrogen levels, which contributes to adverse cardiometabolic changes, including increased blood pressure and visceral obesity, dyslipidemia, impaired glucose metabolism, and insulin resistance [19, 20, 21]. Estrogens are known to regulate key aspects of blood pressure. It enhances endothelial function by promoting nitric oxide production, which facilitates vasodilation and helps maintain vascular tone and also modulates the renin‐angiotensin‐aldosterone system, reduces sympathetic nervous system activity, and has anti‐inflammatory and antioxidant effects [22, 23]. FSH, a gonadotropin hormone secreted by the anterior pituitary, increases markedly during perimenopause and menopause in response to reduced estrogen‐mediated negative feedback [23, 24, 25]. While FSH has traditionally been associated with reproductive function, recent evidence suggests that FSH receptors are expressed in nonreproductive tissues, where it may exert cardiometabolic effects independent of estrogen [11, 12]. FSH potentially contributes to elevated BP in postmenopausal women by promoting renin secretion in the kidneys, influencing lipid metabolism adversely, and interacting with vascular biological changes that collectively increase cardiovascular risk and hypertension. It has shown that FSH receptors are expressed in renal juxtaglomerular cells, which are responsible for renin production. Elevated FSH stimulates renin synthesis via signaling pathways involving protein kinase A, cAMP response element‐binding protein and extracellular signal‐regulated kinase. This stimulation leads to increases in renin, angiotensin II, heart rate, and consequently systolic and diastolic blood pressure in postmenopausal women [17]. Moreover, FSH acts on hepatic FSH receptors to inhibit LDL receptor expression, reducing LDL clearance and leading to dyslipidemia; which are established risk factors for cardiovascular disease and hypertension [26]. However, our study found no association between FSH levels and blood pressure outcomes, suggesting that FSH may not independently contribute to the development of elevated BP or HTN in postmenopausal women.

Previous research has proposed potential links between gonadotropins and cardiovascular risk, hypothesizing that rising FSH levels during the menopausal transition may reflect or even contribute to vascular aging [15, 27, 28, 29].

However, existing evidence regarding the association between serum FSH levels and blood pressure or HTN remains limited and inconclusive. In partial agreement with our findings, Wang et al. (2017) [27] assessed the relationship between FSH and the 10‐year risk of atherosclerotic cardiovascular disease in postmenopausal women. While they found significant associations between FSH and several cardiometabolic risk factors, blood pressure exhibited the weakest association and only marginal statistical significance. This suggests that FSH may not play a major role in modulating blood pressure regulation in postmenopausal women.

In contrast with our findings, Jung et al. (2024) [30] investigated the association between serum FSH levels and cardiometabolic risk factors including BP in post‐menopausal Korean women. A total of 608 post‐menopausal women from eight randomized double‐blind, placebo‐controlled clinical trials on menopause during the year 2012–2019 were analyzed. They reported that SBP was significantly improved as the FSH quartiles increased. The discrepancies between our findings could be attributed to several factors, including differences in study design, population characteristics, and sample size. Notably, Jung et al.'s cohort was derived from clinical trial participants, who may differ from the general population. In contrast, our study draws strength from its large sample size and community‐based design, providing greater external validity to the general population of postmenopausal women.

We hypothesize that the lack of association observed between serum FSH levels and blood pressure outcomes in our study may be due to the possibility that any potential effect of FSH on blood pressure is too small to be detected in population‐based analyses, particularly after adjustment for multiple confounding factors. Additionally, individual variability in FSH receptor sensitivity or expression across tissues [31] may result in heterogeneous vascular responses that dilute overall associations at the population level. Furthermore, it is also plausible that FSH does not have a direct physiological role in modulating vascular tone or blood pressure, and that previous associations reported in the literature were influenced by concurrent hormonal changes during the menopausal transition, such as declining estrogen levels, that more directly impact vascular function. Further studies are warranted to explore these hypotheses, particularly those incorporating mechanistic assessments of FSH signaling pathways in vascular tissues and longitudinal hormone profiling across diverse menopausal populations.

This study has several strengths, including its large, community‐based design, the use of standardized protocols for data collection, and rigorous longitudinal analyses. Nonetheless, certain limitations should be acknowledged. The reliance on a single baseline measurement of FSH may not adequately capture the dynamic changes in FSH levels over time, particularly during the early postmenopausal period when levels can fluctuate significantly before stabilizing. Repeated measurements of FSH across multiple follow‐up visits could have provided a more comprehensive assessment of its long‐term association with elevated BP, HTN, and BP changes. Despite adjustment for a wide range of confounders, residual confounding by unmeasured factors cannot be entirely ruled out. The timing of menopause onset for each participant was not specified, which may introduce heterogeneity; women in early post‐menopause might exhibit different cardiovascular responses to FSH compared to those in later stages, potentially obscuring an association. Additionally, blood pressure in this study was measured twice on the right arm only, rather than three times with initial assessment in both arms as recommended in recent guidelines, which may introduce measurement bias. Finally, the analysis assumed a linear or categorical relationship between FSH and outcome risk, but it did not explore potential nonlinear associations, which might have revealed a more detailed relationship.

In this large, population‐based cohort study of postmenopausal women, we found no significant association between baseline serum FSH levels and the prevalence or incidence of elevated BP and HTN, nor with longitudinal changes in systolic or diastolic blood pressure, Although SBP and DBP changed significantly over time, these trends were not influenced by baseline FSH levels. Our results suggest that serum FSH, despite its established role in reproductive aging, may not be a reliable biomarker for predicting HTN risk or blood pressure changes in postmenopausal women. Further research is warranted to better understand the complex interplay FSH and cardiovascular risk factors including blood pressure.

## Author Contributions

**Fahimeh Ramezani Tehrani:** conceptualization, investigation, funding acquisition, methodology, writing – original draft, formal analysis. **Faegheh Firouzi:** investigation, writing – review and editing, methodology, data curation. **Ramin Farrokhi:** investigation, writing – review and editing, methodology, formal analysis. **Marzieh Saei Ghare Naz:** investigation, writing – review and editing, methodology. **Maryam Mousavi:** investigation, writing – review and editing, writing – original draft, formal analysis. **Fereidoun Azizi:** conceptualization, writing – review and editing, methodology. **Samira Behboudi‐Gandevani:** conceptualization, investigation, formal analysis, writing – original draft, supervision.

## Ethics Statement

The studies involving humans were approved by the ethics committee of the Research Institute for Endocrine Sciences, Shahid Beheshti University of Medical Sciences. Written informed consent was obtained from all subjects. The studies were conducted in accordance with the local legislation and institutional requirements. The participants provided their written informed consent to participate in this study.

## Consent

Informed consent was obtained from all study subjects.

## Conflicts of Interest

The authors declare no conflicts of interest.

## Transparency Statement

The lead author Samira Behboudi‐Gandevani affirms that this manuscript is an honest, accurate, and transparent account of the study being reported; that no important aspects of the study have been omitted; and that any discrepancies from the study as planned (and, if relevant, registered) have been explained.

## Supporting information


**Supplementary Table 1:** The baseline characteristics of participants, stratified by their pre‐hypertension status at follow‐up. **Supplementary Table 2:** The baseline characteristics of participants, stratified by their hypertension status at follow‐up.

## Data Availability

The data that support the findings of this study are available on request from the corresponding author. The data are not publicly available due to privacy or ethical restrictions.

## References

[1] B. Zhou , R. M. Carrillo‐Larco , G. Danaei , et al., “Worldwide Trends in Hypertension Prevalence and Progress in Treatment and Control From 1990 to 2019: A Pooled Analysis of 1201 Population‐Representative Studies With 104 Million Participants,” Lancet 398 (2021): 957–980. 34450083 10.1016/S0140-6736(21)01330-1 PMC8446938

[2] J. E. Sharman , E. O'Brien , B. Alpert , et al., “Lancet Commission on Hypertension Group Position Statement on the Global Improvement of Accuracy Standards for Devices That Measure Blood Pressure,” Journal of Hypertension 38 (2020): 21–29. 31790375 10.1097/HJH.0000000000002246 PMC6919228

[3] K. T. Mills , A. Stefanescu , and J. He , “The Global Epidemiology of Hypertension,” Nature Reviews Nephrology 16 (2020): 223–237. 32024986 10.1038/s41581-019-0244-2 PMC7998524

[4] R. Lima , M. Wofford , and J. F. Reckelhoff , “Hypertension in Postmenopausal Women,” Current Hypertension Reports 14 (2012): 254–260. 22427070 10.1007/s11906-012-0260-0 PMC3391725

[5] L. Ghazi , R. V. Annabathula , N. A. Bello , L. Zhou , R. B. Stacey , and B. Upadhya , “Hypertension Across a Woman's Life Cycle,” Current Hypertension Reports 24 (2022): 723–733. 36350493 10.1007/s11906-022-01230-4 PMC9893311

[6] S. Taddei , “Blood Pressure Through Aging and Menopause,” supplement, Climacteric 12, no. S1 (2009): 36–40. 19811239 10.1080/13697130903004758

[7] B. N. Okeahialam , H. Agbo , E. Chuhwak , and I. Isiguzoro , “Arterial Hypertension in Women: Menopause as a Risk Window,” Post Reproductive Health 28 (2022): 19–22. 34889118 10.1177/20533691211063342

[8] M. Lispi , P. Humaidan , G. R. Bousfield , T. D'Hooghe , and A. Ulloa‐Aguirre , “Follicle‐Stimulating Hormone Biological Products: Does Potency Predict Clinical Efficacy?,” International Journal of Molecular Sciences 24 (2023): 9020. 37240364 10.3390/ijms24109020 PMC10218858

[9] C. Taneja , S. Gera , S. M. Kim , J. Iqbal , T. Yuen , and M. Zaidi , “FSH‐Metabolic Circuitry and Menopause,” Journal of Molecular Endocrinology 63 (2019): R73–R80. 31454787 10.1530/JME-19-0152 PMC6992500

[10] R. Costa , T. P. Tuomainen , J. Virtanen , L. Niskanen , and E. Bertone‐Johnson , “Associations of Reproductive Factors With Postmenopausal Follicle Stimulating Hormone,” Women's Midlife Health 8 (2022): 8. 36059005 10.1186/s40695-022-00079-6 PMC9442942

[11] C. Li , Y. Ling , and H. Kuang , “Research Progress on FSH‐FSHR Signaling in the Pathogenesis of Non‐Reproductive Diseases,” Frontiers in Cell and Developmental Biology 12 (2024): 1506450. 39633710 10.3389/fcell.2024.1506450 PMC11615068

[12] X. M. Liu , H. C. Chan , G. L. Ding , et al., “FSH Regulates Fat Accumulation and Redistribution in Aging Through the Gαi/Ca(2+)/CREB Pathway,” Aging Cell 14 (2015): 409–420. 25754247 10.1111/acel.12331 PMC4406670

[13] X. Li , W. Chen , P. Li , et al., “Follicular Stimulating Hormone Accelerates Atherogenesis by Increasing Endothelial VCAM‐1 Expression,” Theranostics 7 (2017): 4671–4688. 29187895 10.7150/thno.21216 PMC5706091

[14] H. Zhu , G. Ding , and H. Huang , “FSH Regulates Glucose‐Stimulated Insulin Secretion: A Bell‐Shaped Curve Effect,” Journal of Diabetes 16 (2024): e13546. 38599851 10.1111/1753-0407.13546 PMC11006606

[15] C. Serviente , T. P. Tuomainen , J. Virtanen , S. Witkowski , L. Niskanen , and E. Bertone‐Johnson , “Follicle‐Stimulating Hormone Is Associated With Lipids in Postmenopausal Women,” Menopause 26 (2019): 540–545. 30562316 10.1097/GME.0000000000001273 PMC6483826

[16] N. Lin , K. C. van Zomeren , T. Plosch , et al., “Follicle‐Stimulating Hormone Stimulates Free Radical Generation Without Inducing Substantial Oxidative Stress in Human Granulosa Cells,” Human Reproduction Open 2025 (2025): hoaf007. 40066298 10.1093/hropen/hoaf007 PMC11893154

[17] Z. Yu , J. Yang , W. J. Huang , et al., “Follicle Stimulating Hormone Promotes Production of Renin Through Its Receptor in Juxtaglomerular Cells of Kidney,” Diabetology & Metabolic Syndrome 14 (2022): 65. 35501878 10.1186/s13098-022-00816-x PMC9063271

[18] P. K. Whelton , R. M. Carey , W. S. Aronow , et al., “2017 ACC/AHA/AAPA/ABC/ACPM/AGS/APhA/ASH/ASPC/NMA/PCNA Guideline for the Prevention, Detection, Evaluation, and Management of High Blood Pressure in Adults: Executive Summary: A Report of the American College of Cardiology/American Heart Association Task Force on Clinical Practice Guidelines,” Hypertension 71 (2018): 1269–1324. 29133354 10.1161/HYP.0000000000000066

[19] F. Mauvais‐Jarvis , D. J. Clegg , and A. L. Hevener , “The Role of Estrogens in Control of Energy Balance and Glucose Homeostasis,” Endocrine Reviews 34 (2013): 309–338. 23460719 10.1210/er.2012-1055 PMC3660717

[20] R. Prabhushankar , C. Krueger , and C. Manrique , “Membrane Estrogen Receptors: Their Role in Blood Pressure Regulation and Cardiovascular Disease,” Current Hypertension Reports 16 (2014): 408. 24343167 10.1007/s11906-013-0408-6

[21] B. Visniauskas , I. Kilanowski‐Doroh , B. O. Ogola , et al., “Estrogen‐Mediated Mechanisms in Hypertension and Other Cardiovascular Diseases,” Journal of Human Hypertension 37 (2023): 609–618. 36319856 10.1038/s41371-022-00771-0 PMC10919324

[22] A. Scuteri and L. Ferrucci , “Blood Pressure, Arterial Function, Structure, and Aging: The Role of Hormonal Replacement Therapy in Postmenopausal Women,” Journal of Clinical Hypertension 5 (2003): 219–225. 12826785 10.1111/j.1524-6175.2003.00683.x PMC8101833

[23] A. Allshouse , J. Pavlovic , and N. Santoro , “Menstrual Cycle Hormone Changes Associated With Reproductive Aging and How They May Relate to Symptoms,” Obstetrics and Gynecology Clinics of North America 45 (2018): 613–628. 30401546 10.1016/j.ogc.2018.07.004 PMC6226272

[24] N. Santoro , C. Roeca , B. A. Peters , and G. Neal‐Perry , “The Menopause Transition: Signs, Symptoms, and Management Options,” Journal of Clinical Endocrinology & Metabolism 106 (2021): 1–15. 33095879 10.1210/clinem/dgaa764

[25] C. K. Welt , “Female Reproductive Aging Is Marked by Decreased Secretion of Dimeric Inhibin,” Journal of Clinical Endocrinology & Metabolism 84 (1999): 105–111. 9920069 10.1210/jcem.84.1.5381

[26] Y. Song , E. S. Wang , L. L. Xing , et al., “Follicle‐Stimulating Hormone Induces Postmenopausal Dyslipidemia Through Inhibiting Hepatic Cholesterol Metabolism,” Journal of Clinical Endocrinology & Metabolism 101 (2016): 254–263. 26583582 10.1210/jc.2015-2724

[27] N. Wang , H. Shao , Y. Chen , et al., “Follicle‐Stimulating Hormone, Its Association With Cardiometabolic Risk Factors, and 10‐Year Risk of Cardiovascular Disease in Postmenopausal Women,” Journal of the American Heart Association 6 (2017): e005918. 28855169 10.1161/JAHA.117.005918 PMC5634260

[28] S. W. Lee , I. S. Hwang , G. Jung , H. J. Kang , and Y. H. Chung , “Relationship Between Metabolic Syndrome and Follicle‐Stimulating Hormone in Postmenopausal Women,” Medicine 101 (2022): e29216. 35550473 10.1097/MD.0000000000029216 PMC9276200

[29] Y. Chen , C. Wang , B. Sun , et al., “Associations of Follicle‐Stimulating Hormone and Luteinizing Hormone With Metabolic Syndrome During the Menopausal Transition From the National Health and Nutrition Examination Survey,” Front Endocrinol (Lausanne) 14 (2023): 1034934. 36843613 10.3389/fendo.2023.1034934 PMC9947143

[30] E. S. Jung , E. K. Choi , B. H. Park , and S. W. Chae , “Serum Follicle‐Stimulating Hormone Levels Are Associated With Cardiometabolic Risk Factors in Post‐Menopausal Korean Women,” Journal of Clinical Medicine 9 (2020): 1161. 32325717 10.3390/jcm9041161 PMC7230188

[31] D. Sun , M. Bai , Y. Jiang , et al., “Roles of Follicle Stimulating Hormone and Its Receptor in Human Metabolic Diseases and Cancer,” American Journal of Translational Research 12 (2020): 3116–3132. 32774689 PMC7407683

