# Supplementary material for: Association Between Serum Follicle‐Stimulating Hormone Levels and Risk of Elevated Blood Pressure and Hypertension Among Postmenopausal Women: A Longitudinal Population‐Based Study
Source: Health Sci Rep. 2025 Oct 15;8(10):e71325. doi: 10.1002/hsr2.71325 (PMC12528801; doi:10.1002/hsr2.71325)
Supplement: Supplementary file 1 — Supplementary Table 1: The baseline characteristics of participants, stratified by their pre‐hypertension status at follow‐up. Supplementary Table 2: The baseline characteristics of participants, stratified by their hypertension status at follow‐up. [file HSR2-8-e71325-s001.docx]

Supplementary material

Supplementary Table 1. The baseline characteristics of participants, stratified by their pre-hypertension status at follow-up.

| Variable | | Overall (n = 221) | Healthy (n = 87) | Pre-HTN (n = 134) | P-value |
| --- | --- | --- | --- | --- | --- |
| Age (years), Mean (SD) | | 56.30 (6.52) | 57.00 (6.75) | 55.85 (6.36) | 0.20 |
| BMI, Mean (SD) | | 29.28 (3.94) | 29.21 (4.40) | 29.33 (3.62) | 0.82 |
| Total cholesterol, Mean (SD) | | 222.86 (42.02) | 228.22 (43.87) | 219.38 (40.55) | 0.13 |
| Systolic BP, Mean (SD) | | 107.43 (8.02) | 105.55 (9.05) | 108.64 (7.05) | 0.005 |
| Diastolic BP, Mean (SD) | | 69.82 (6.15) | 69.01 (6.24) | 70.35 (6.05) | 0.11 |
| HDL cholesterol, Median [IQR] | | 42.00 [35.00, 49.00] | 42.00 [35.00, 49.00] | 39.50 [35.00, 49.00] | 0.31 |
| TG, Median [IQR] | | 153.00 [104.00, 210.00] | 153.00 [106.50, 209.50] | 153.00 [104.00, 211.50] | 0.93 |
| Parity, Mean (SD) | | 4.60 (1.85) | 4.75 (2.08) | 4.50 (1.70) | 0.34 |
| Menopause age, Mean (SD) | | 49.72 (5.59) | 50.32 (4.82) | 49.33 (6.03) | 0.20 |
| Family history of HTN, N (%) | |  |  |  | 0.59 |
|  | No | 206 (93.6%) | 80 (92.0%) | 126 (94.7%) |  |
|  | Yes | 14 (6.4%) | 7 (8.0%) | 7 (5.3%) |  |
| Physical activity, N (%) | |  |  |  | 0.33 |
|  | No | 69 (31.7%) | 31 (36.0%) | 38 (28.8%) |  |
|  | yes | 149 (68.3%) | 55 (64.0%) | 94 (71.2%) |  |
| Smoking status, N (%) | |  |  |  | 0.17 |
|  | Non-smoker | 205 (94.9%) | 78 (91.8%) | 127 (96.9%) |  |
|  | Smoker | 11 (5.1%) | 7 (8.2%) | 4 (3.1%) |  |

Supplementary Table 2. The baseline characteristics of participants, stratified by their hypertension status at follow-up.

| Variable | | Overall (n = 500) | Not - HTN (n = 205) | Hypertensive (n = 295) | P-value |
| --- | --- | --- | --- | --- | --- |
| Age (years), Mean (SD) | | 57.13 (6.60) | 56.28 (7.03) | 57.73 (6.23) | 0.02 |
| BMI, Mean (SD) | | 29.66 (4.28) | 29.12 (4.12) | 30.03 (4.36) | 0.02 |
| Total Cholesterol, Mean (SD) | | 222.40 (41.99) | 218.19 (40.13) | 225.34 (43.06) | 0.06 |
| Systolic BP, Mean (SD) | | 117.65 (12.16) | 113.06 (11.86) | 120.83 (11.35) | <0.001 |
| Diastolic BP, Mean (SD) | | 75.15 (7.91) | 72.76 (7.86) | 76.82 (7.51) | <0.001 |
| HDL Cholesterol, Median [IQR] | | 40.00 [35.00, 49.00] | 42.00 [35.00, 49.00] | 39.00 [35.00, 47.00] | 0.57¹ |
| Triglycerides, Median [IQR] | | 159.00 [117.5, 219.0] | 155.00 [107.0, 215.0] | 163.50 [120.0, 220.0] | 0.14¹ |
| Parity, Mean (SD) | | 4.78 (1.97) | 4.47 (1.93) | 4.99 (1.98) | 0.004 |
| Menopause Age, Mean (SD) | | 49.89 (5.60) | 49.08 (6.14) | 50.46 (5.13) | 0.007 |
| Family History of HTN (%) | |  |  |  | 0.01 |
|  | No | 423 (85.3%) | 184 (90.2%) | 239 (81.8%) |  |
|  | Yes | 73 (14.7%) | 20 (9.8%) | 53 (18.2%) |  |
| Physical Activity (%) | |  |  |  | >0.99 |
|  | No | 158 (31.9%) | 65 (32.2%) | 93 (31.7%) |  |
|  | Yes | 337 (68.1%) | 137 (67.8%) | 200 (68.3%) |  |
| Smoking Status (%) | |  |  |  | 0.47 |
| Non-smoker | | 469 (95.1%) | 189 (94.0%) | 280 (95.9%) |  |
| Smoker | | 24 (4.9%) | 12 (6.0%) | 12 (4.1%) |  |
